# Effect of food-simulating liquids on flexural strength, Vickers microhardness, and surface roughness of IPS e.max Press and CEREC Tessera

**DOI:** 10.2340/biid.v13.46275

**Published:** 2026-06-23

**Authors:** Asmaa Abdeewi, Seham A. Elsawaay, Ahmed A. Mhanni, Abubaker Qutieshat

**Affiliations:** a Libyan Polymer Research Center, Tripoli, Libya; b Research and Consultancy Center, Sirte University, Tripoli, Libya; c Fixed Prosthodontics Department, Faculty of Dentistry, University of Tripoli, Tripoli, Libya; d Restorative Dentistry, College of Dental Medicine, University of Sharjah, Sharjah, UAE

**Keywords:** Ceramics, citric acid, ethanol, saliva, artificial, flexural strength, mechanical tests, surface properties

## Abstract

**Background:**

Lithium disilicate ceramics are widely used for indirect restorations, yet their mechanical performance may be influenced by fabrication route and chemical challenge from foods and beverages.

**Objective:**

To compare the effects of food-simulating liquids (FSLs) on flexural strength, Vickers microhardness, and surface roughness of pressed lithium disilicate (IPS e.max Press) and Computer-Aided Design and Computer-Aided Manufacturing (CAD/CAM) advanced lithium disilicate (CEREC Tessera).

**Materials and methods:**

One hundred fifty rectangular specimens were prepared using IPS e.max Press and CEREC Tessera (*n* = 75 each) and allocated into five groups per material (*n* = 15): one unconditioned control group, artificial saliva (7 days at 37°C), 0.02% citric acid, 20% ethanol, or 50% ethanol. Citric acid and ethanol groups underwent three 5-minute challenge cycles followed by re-storage in artificial saliva. Flexural strength was assessed by three-point bending. Microhardness (Vickers HV1/10) and surface roughness (Ra) were measured on independent specimens. Data were analysed using two-way analysis of variance with Tukey’s post hoc test for flexural strength and surface roughness. For microhardness, factorial analysis was limited to the comparable shared conditions because ethanol-exposed IPS e.max Press specimens did not yield reliable Vickers measurements.

**Results:**

CEREC Tessera showed higher flexural strength than IPS e.max Press across conditions (control: 362.6 ± 15.4 vs 331.0 ± 8.5 MPa). Ethanol reduced strength in both materials, with the greatest reduction in 50% ethanol (238.0 ± 9.6 vs 147.4 ± 7.5 MPa). Across comparable conditions, CEREC Tessera also showed higher microhardness, while reliable Vickers measurements could not be obtained for ethanol-exposed IPS e.max Press specimens. Surface roughness increased after acidic and alcoholic exposure in both materials, although CEREC Tessera remained smoother overall (control: 0.06 ± 0.02 vs 0.67 ± 0.36 µm).

**Conclusion:**

CEREC Tessera demonstrated superior flexural strength, microhardness, and surface integrity compared with IPS e.max Press after exposure to FSLs, suggesting that fabrication route influences laboratory performance under chemical challenge.

## Introduction

Lithium disilicate ceramics are widely used in restorative dentistry because of their favourable mechanical properties, aesthetics, and biocompatibility. Their clinical success depends on intrinsic material properties and on the ability to withstand the chemical challenges of the oral environment, including exposure to foods and beverages [1]. IPS e.max Press and CEREC Tessera represent two leading lithium disilicate-based restorative materials. IPS e.max Press is a pressable lithium disilicate glass-ceramic, valued for its strength, aesthetics and versatility in indirect restorations such as crowns, veneers, and inlays/onlays. CEREC Tessera is a more recent lithium silicate glass-ceramic designed for chairside CAD/CAM workflows, offering rapid crystallization and high aesthetic potential. Both materials are indicated for single-unit restorations and, in some cases, short-span fixed dental prostheses, with clinical longevity hinging on mechanical robustness and surface integrity, which are key determinants of durability, aesthetics, and biocompatibility in the oral environment [2–5].

In general, IPS e.max Press has been reported to exhibit higher fracture toughness and flexural strength than CEREC Tessera [2, 3, 6–8]. However, IPS e.max Press appears more sensitive to certain surface treatments (e.g. hydrofluoric acid etching) and may show greater degradation under fatigue loading and repeated processing cycles [9, 10]. By comparison, CEREC Tessera, despite lower absolute strength in some reports, may maintain more stable mechanical behaviour under cyclic loading, present smoother surfaces, and demonstrate favourable wear characteristics and enamel friendliness [4, 8, 11, 12].

Food-simulating liquids (FSLs) are commonly used in vitro to approximate chemical challenges associated with dietary and oral exposure, allowing the effects of aqueous, acidic, and alcohol-related media on restorative materials to be investigated under controlled conditions [1, 13]. Artificial saliva or water is commonly used to simulate moist salivary exposure, citric acid to represent acidic dietary challenge, and ethanol to simulate alcohol-containing liquids [1, 13]. Previous studies have shown that exposure to FSLs or erosive media can alter the surface and mechanical properties of lithium disilicate and related glass-ceramics, including hardness, flexural strength, surface roughness, colour stability, and ion release, with the magnitude of change influenced by pH, exposure time, temperature, material composition, crystalline microstructure, and surface finishing [1, 13–17]. Acidic media are of particular relevance because they may promote glass-matrix degradation, alkaline-ion leaching, and surface irregularities, while finishing procedures such as glazing, polishing, or silanization may modify the extent of surface degradation [1, 13, 15, 17].

Although these studies provide important evidence on the chemical ageing of dental ceramics, the available literature has generally addressed CAD/CAM lithium disilicate systems, partially versus fully crystallized ceramics, thermocycling, acidic challenge, or surface degradation separately [1, 13–17]. Direct evidence remains limited regarding the behaviour of pressed lithium disilicate and CAD/CAM advanced lithium disilicate when tested under the same FSL conditions using a combined mechanical and surface-property framework [1, 17]. In particular, it remains insufficiently understood whether fabrication route and material microstructure influence the combined behaviour of flexural strength, Vickers microhardness, and surface roughness after exposure to media simulating salivary, acidic, and alcohol-related dietary challenges [1, 18]. Flexural strength was selected as an indicator of resistance to fracture under bending stress, Vickers microhardness as a measure of surface resistance to indentation and localized degradation, and surface roughness as a clinically relevant marker of surface integrity that may influence plaque retention, staining, antagonist wear, and restoration maintenance [19]. This study therefore investigated the effects of FSLs on IPS e.max Press and CEREC Tessera, representing pressed and CAD/CAM lithium disilicate-based systems, respectively. The null hypothesis was that no significant differences in these properties would be observed between the two ceramics after exposure to FSLs.

## Materials and methods

Institutional clearance for this *in vitro* laboratory study was obtained from the Research Ethics Committee of the Polymer Research Centre (LPRC) (Ref. LPRC/6/729).

### Materials and sample preparation

This study evaluated two ceramic materials: IPS e.max Press (Ivoclar Vivadent, Lot# Z02G42, CE0123) and CEREC Tessera (Dentsply Sirona, Lot# 16013293). IPS e.max Press is supplied as a pressable lithium disilicate ingot for the heat-pressing technique, whereas CEREC Tessera is supplied as an advanced lithium disilicate CAD/CAM block requiring a post-milling firing cycle, with or without glaze, to achieve its intended mechanical properties (Table 1).

**Table 1:** Materials used in the study.

Trade name	Type	Composition
IPS e.max Press	Lithium disilicate glass-ceramic	SiO₂: 57–80 wt%; Li₂O: 11–19 wt%; K₂O: 0–13 wt%; P₂O₅: 0–11 wt%; ZrO₂: 0–8 wt%; ZnO: 0–8 wt%; other oxides and ceramic pigments: 0–10 wt%
CEREC Tessera	Advanced lithium disilicate glass-ceramic	Lithium disilicate (Li_2_Si_2_O_5_), with virgilite formed during firing in a zirconia-enriched glass matrix; additional oxides include K_2_O, MgO, ZnO, Al_2_O_3_, and P_2_O_5_

Seventy-five IPS e.max Press specimens were fabricated using the heat-pressing technique. Wax sheets were cut into rectangular specimens (18 × 15 × 1 mm), invested, placed in silicone rings, preheated, and pressed according to the manufacturer’s recommendations. After divestment, the pressed specimens were cleaned, air-dried, and the sprues were removed. The surfaces were then air-abraded with Al_2_O_3_, steam-cleaned, and finished according to the manufacturer’s instructions. Final surface treatment was performed using IPS Ivocolor Glaze Paste (Ivoclar Vivadent AG, Schaan, Liechtenstein), which was applied in paste form with a brush, followed by one glaze-firing cycle. According to Ivoclar’s firing program for IPS e.max Press with IPS Ivocolor, glaze firing was performed at a final temperature of 710°C with a 1-minute holding time.

Similarly, 75 CEREC Tessera specimens were obtained by sectioning CAD/CAM blocks using a 5-axis water jet cutting machine (ACCURL, Model: MAX-WJ-2030, Anhui, China) to produce rectangular specimens (18 × 15 × 1 mm). After sectioning, the specimens were polished using abrasive discs and cleaned before glazing. A thin, even glaze layer was then applied to the external surfaces using CEREC Tessera glaze material (DentSply Sirona, USA) in accordance with the manufacturer’s processing instructions, and the specimens underwent the manufacturer-recommended mandatory matrix/glaze firing cycle in a ceramic furnace to achieve the intended mechanical properties. According to the CEREC Tessera processing guide, glaze firing is performed using the designated glaze program with a final temperature of 760°C and a 2-minute holding time. All specimens were processed in accordance with the respective manufacturers’ instructions.

### Artificial saliva formulation

Artificial saliva was prepared in-house according to the Fusayama formulation [20]. The solution was prepared in deionized water using sodium chloride (NaCl), potassium chloride (KCl), sodium dihydrogen phosphate (NaH_2_PO_4_.H_2_O), calcium chloride dihydrate (CaCl_2_.2H_2_O), sodium sulphide nonahydrate (Na_2_S.9H_2_O), and urea (CH_4_N_2_O), as summarized in Table 2. All immersion procedures were conducted in glass containers to standardize exposure conditions.

**Table 2:** FSLs used in the study.

FSL	Composition	Common name	Manufacturer
Artificial saliva	400 mg/L NaCl 400 mg/L KCl 795 mg/L CaCl_2_.2H_2_O 690 mg/L NaH_2_PO_4_.H_2_O 5 mg/L Na_2_S.9H_2_O 1000 mg/L Urea (CH_4_N_2_O)	Fusayama solution	Prepared in-house
Ethanol	C_2_H_5_OH	Ethyl alcohol	Razi, Iran
Citric acid	C_6_H_8_O_7_	Citric acid solution	Dr. Mojallali, Iran

FSL: food-simulating liquids.

### FSL immersion protocol

One hundred fifty specimens were prepared, comprising 75 IPS e.max Press and 75 CEREC Tessera specimens. For each material, specimens were randomly allocated into five groups (*n* = 15 per group): one unconditioned control group and four FSL exposure groups. The unconditioned control specimens were not exposed to any FSL and were tested after completion of specimen preparation, glazing/firing, cleaning, and drying. They were kept dry in closed containers at room temperature until testing. The exposure groups were artificial saliva stored at 37°C for 7 days, 0.02% citric acid, 20% ethanol, and 50% ethanol. For the citric acid and ethanol groups, specimens underwent three cycles of 5-minute immersion in the assigned solution, followed by re-storage in artificial saliva, as shown in Table 2. After completion of the assigned condition, specimens were rinsed with saline, air-dried, and allocated across the three outcome measures: flexural strength, microhardness, and surface roughness. The five-group design was selected to allow comparison between a baseline unconditioned control, salivary storage, acidic challenge, and alcohol-related food-simulating challenge under balanced material x condition groups. Artificial saliva was included as a controlled aqueous medium that approximates salivary ionic exposure, while citric acid was selected to represent acidic dietary challenge commonly associated with erosive foods and beverages. Ethanol at 20 and 50% was included as an alcohol-related food-simulating medium to evaluate whether increasing alcohol concentration affected the tested ceramic properties. The cyclic exposure protocol followed by re-storage in artificial saliva was adopted to model intermittent chemical challenge rather than continuous immersion, in line with laboratory approaches used to approximate repeated short-term dietary exposures [21, 22].

### Experimental design

**Figure 1:**
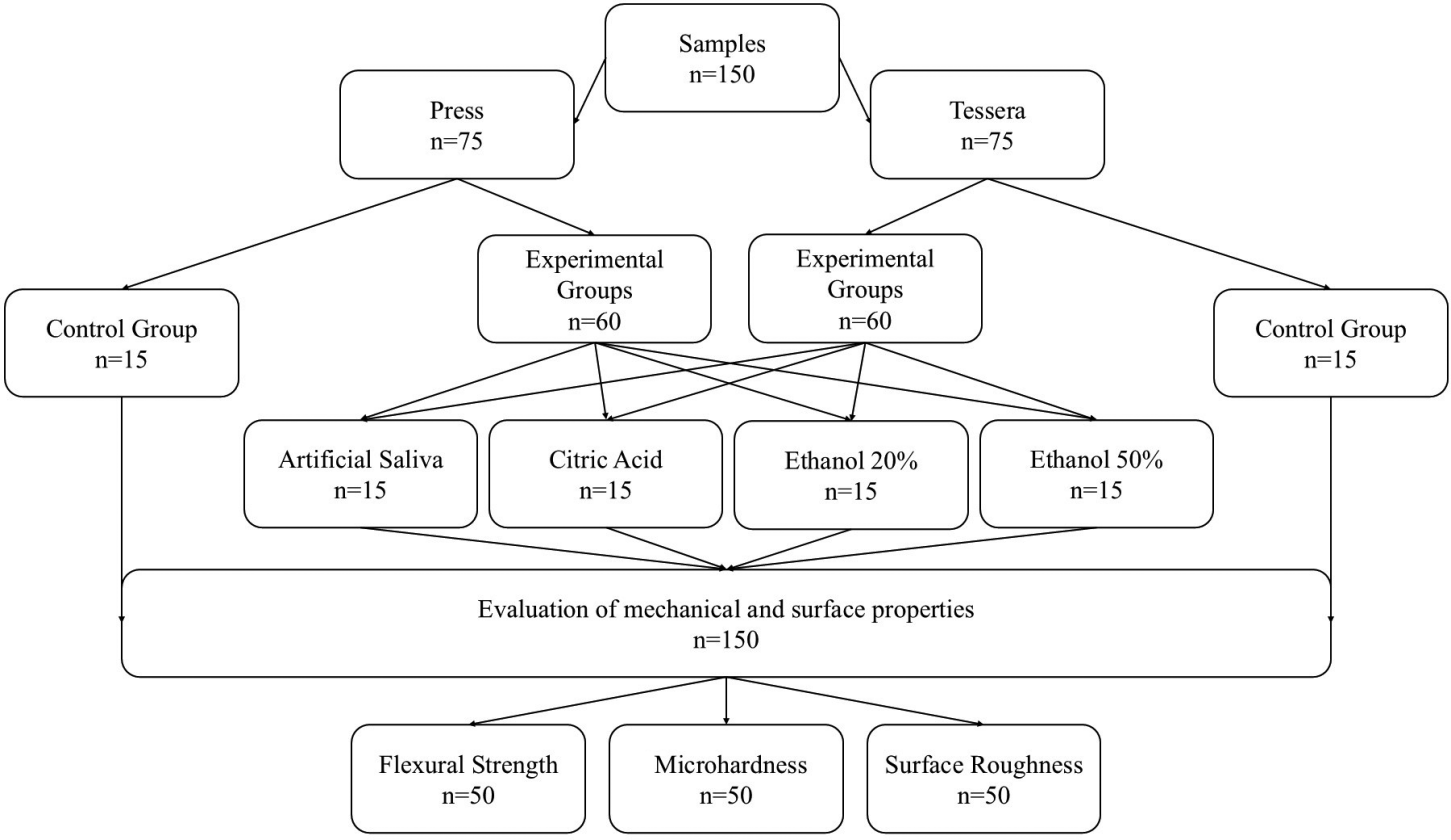
Allocation of 150 lithium disilicate-based ceramic specimens (IPS e.max Press, *n* = 75; CEREC Tessera, *n* = 75) into five groups per material (*n* = 15 per group): one unconditioned control group and four FSL exposure groups comprising artificial saliva, 0.02 citric acid, 20 ethanol, and 50% ethanol. From each material × condition group (*n* = 15), specimens were allocated across the three outcomes (flexural strength, microhardness and surface roughness), resulting in *n* = 5 per outcome and a total of 50 specimens per test. FSL: food-simulating liquids.

Samples were randomly allocated into five groups per material. One group served as an unconditioned control, in which specimens received no FSL exposure and were kept dry in closed containers at room temperature until testing. The remaining four groups were assigned to artificial saliva (7 days at 37°C), 0.02% citric acid, 20% ethanol, or 50% ethanol, as described above. After completion of the assigned condition, all specimens were rinsed with saline, air-dried, and subjected to the assigned outcome testing (Figure 1).

### Flexural strength testing

A three-point bending test was performed using a universal testing machine (Instron 4411, Massachusetts, USA) in accordance with ISO 6872:2024 (Dentistry – Ceramic materials) [23]. Specimens were tested over a support span of 12 mm at a crosshead speed of 0.5 mm/min. Each specimen was positioned centrally on the two supports and loaded at the midpoint until fracture. The maximum fracture load (P, N) was recorded for each specimen. Flexural strength (S, MPa) was calculated using the standard equation for rectangular specimens:


$$S=\frac{3PL}{2WH^{2}}$$

where P is the maximum applied load (N), L is the support span (mm), W is the specimen width (mm), and H is the specimen thickness/height (mm). Specimen dimensions were verified prior to testing using a digital calliper.

### Microhardness testing

Micro-Vickers hardness values were determined using a digital microhardness tester (TIV GE Inspection Technologies). A 1 kgf load was applied for 10 seconds (HV1/10) to each specimen. Five independent specimens per material per FSL condition (*n* = 5) were tested. For each specimen, three indentations were placed on the tested surface at separated locations away from specimen edges, and the mean Vickers hardness value was calculated per specimen. Group means and standard deviations were then computed from the specimen-level means. Hardness assessment was based on standardized Vickers indentation principles consistent with ISO 6507-1:2018 and ASTM C1327-15 [24, 25].

### Surface roughness testing

Surface roughness was measured using a Roughness Tester (PCE-RT 2000). A stylus traversed each sample’s surface, and Ra values were recorded. Five independent specimens per material per FSL condition (*n* = 5) were assessed. Three traces were recorded at different locations on each specimen surface, and the three Ra readings were averaged to obtain a single Ra value per specimen. Group means and standard deviations were calculated from specimen-level averages.

### Statistical analysis

Data were analysed using IBM SPSS Statistics version 26.0 (IBM Corp.). Descriptive statistics were expressed as means and standard deviations. Assumptions of normality and homogeneity of variance were assessed using the Shapiro–Wilk and Levene tests, respectively. For flexural strength and surface roughness, a two-way analysis of variance (ANOVA) was performed to assess the main effects of ceramic material and food-simulating solution, as well as their interaction. Tukey’s post hoc test was used for pairwise multiple comparisons where applicable. For microhardness, full factorial analysis across all five solution conditions was not feasible because reliable Vickers measurements could not be obtained for IPS e.max Press specimens exposed to 20 and 50% ethanol. Therefore, inferential microhardness analysis was limited to the comparable conditions shared by both materials (control, artificial saliva, and citric acid). A significance level of *p* < 0.05 was adopted for all analyses. Effect sizes for ANOVA models were reported as partial eta squared (ηp^2^) to complement statistical significance testing.

## Results

**Figure 2:**
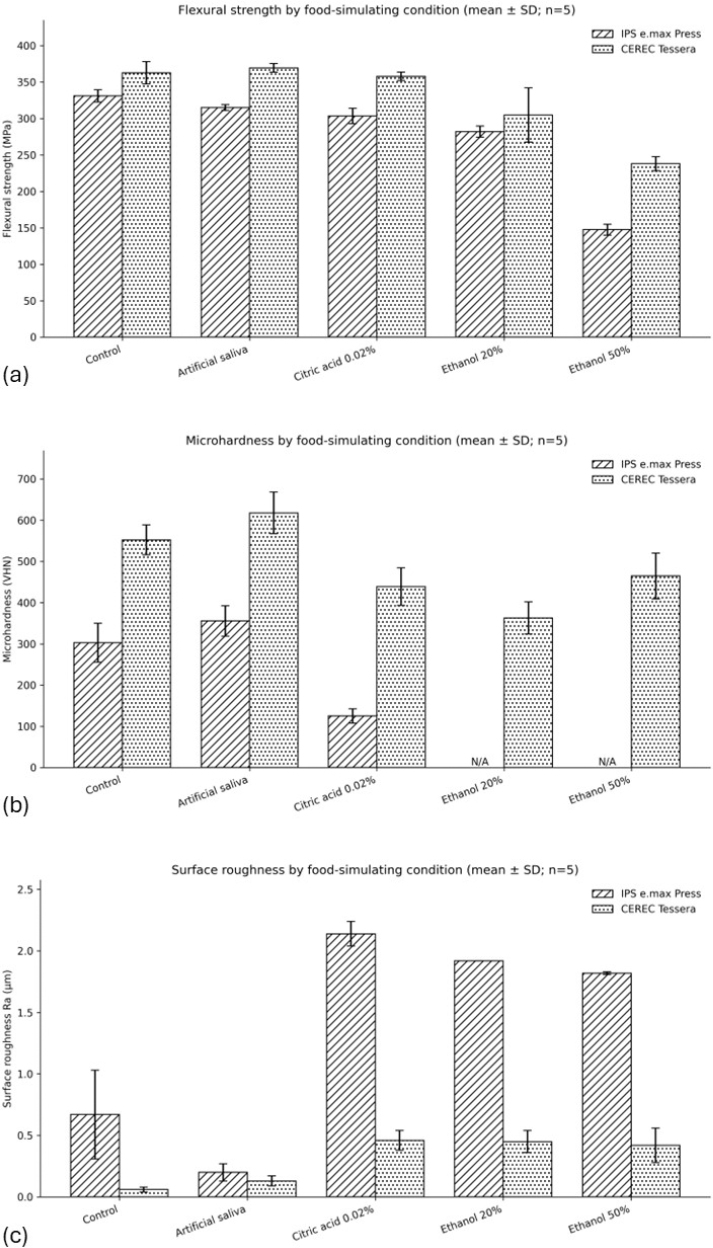
Mechanical properties of IPS e.max Press and CEREC Tessera after exposure to FSLs. (a) Flexural strength (MPa). (b) Vickers microhardness (VHN). (c) Surface roughness (Ra, µm). Bars show mean ± SD (*n* = 5 independent specimens per material per condition). FSL conditions were control, artificial saliva, 0.02 citric acid, 20 ethanol, and 50% ethanol. Microhardness values for IPS e.max Press in the ethanol groups were not measurable due to post-exposure surface condition that precluded reliable Vickers indentation. FSL: food-simulating liquids.

Descriptive statistics for flexural strength, microhardness, and surface roughness across all material and FSL conditions are presented in Table 3 and illustrated in Figure 2a–c. Factorial inferential analyses are summarized in Table 4.

**Table 3:** Descriptive statistics for flexural strength, microhardness, and surface roughness of IPS e.max Press and CEREC Tessera across FSL conditions.

Material	FSL	Flexural strength (MPa), mean ± SD	Microhardness (VHN), mean ± SD	Surface roughness (µm), mean ± SD
IPS e.max Press	Control	331.0 ± 8.5	302.6 ± 47.3	0.67 ± 0.36
	Artificial saliva	315.0 ± 4.1	355.6 ± 36.8	0.20 ± 0.07
	Citric acid 0.02%	303.4 ± 10.6	125.0 ± 17.3	2.14 ± 0.10
	Ethanol 20%	282.0 ± 7.6	Not measurable	1.92 ± 0.00
	Ethanol 50%	147.4 ± 7.5	Not measurable	1.82 ± 0.01
CEREC Tessera	Control	362.6 ± 15.4	552.4 ± 36.4	0.06 ± 0.02
	Artificial saliva	369.4 ± 5.9	617.8 ± 50.5	0.13 ± 0.04
	Citric acid 0.02%	357.8 ± 5.9	439.0 ± 45.2	0.46 ± 0.08
	Ethanol 20%	304.8 ± 37.3	362.8 ± 39.0	0.45 ± 0.09
	Ethanol 50%	238.0 ± 9.6	464.8 ± 55.7	0.42 ± 0.14

*n* = 5 independent specimens per material per condition for each outcome. FSL: food-simulating liquids.

**Table 4:** Factorial statistical analysis of the effects of ceramic material and FSL on flexural strength, microhardness, and surface roughness.

Outcome	Analysis scope	Effect	*df*	*F*	*P*	ηp^2^
Flexural strength	Full 2×5 factorial design	Material	1.40	153.31	< 0.001	0.793
		FSL	4.40	195.85	< 0.001	0.951
		Material × liquid interaction	4.40	8.22	< 0.001	0.451
Microhardness	Restricted 2×3 factorial design*	Material	1.24	347.79	< 0.001	0.935
		FSL	2.24	67.88	< 0.001	0.850
		Material × liquid interaction	2.24	1.77	0.191	0.129
Surface roughness	Full 2×5 factorial design	Material	1.40	756.86	< 0.001	0.950
		FSL	4.40	150.72	< 0.001	0.938
		Material × liquid interaction	4.40	63.98	< 0.001	0.865

* Microhardness factorial analysis was limited to the comparable shared conditions of control, artificial saliva, and 0.02% citric acid because ethanol-exposed IPS e.max Press specimens did not yield reliable Vickers measurements. Tukey’s post hoc test was used for pairwise comparisons where applicable. FSL: food-simulating liquids.

For flexural strength, two-way ANOVA showed significant main effects of ceramic material and FSL, as well as a significant material × liquid interaction (material: *F*(1.40) = 153.31, *p* < 0.001; liquid: *F*(4.40) = 195.85, *p* < 0.001; interaction: *F*(4.40) = 8.22, *p* < 0.001). Overall, CEREC Tessera demonstrated higher flexural strength than IPS e.max Press, while exposure to FSLs reduced strength in a solution-dependent manner. Descriptively, IPS e.max Press showed a progressive decline in flexural strength with ethanol exposure, with the greatest reduction observed in 50% ethanol, whereas CEREC Tessera remained relatively stable in artificial saliva and citric acid and showed lower values mainly in the ethanol groups (Table 3; Figure 2a).

For microhardness, a complete factorial analysis across all five liquid conditions was not possible because reliable Vickers measurements could not be obtained for IPS e.max Press specimens exposed to 20 and 50% ethanol. Inferential analysis was therefore restricted to the comparable shared conditions of control, artificial saliva, and citric acid. Within these conditions, two-way ANOVA showed significant main effects of ceramic material and FSL, while the interaction was not statistically significant (material: *F*(1.24) = 347.79, *p* < 0.001; liquid: *F*(2.24) = 67.88, *p* < 0.001; interaction: *F*(2.24) = 1.77, *p* = 0.191). Across the comparable conditions, CEREC Tessera maintained higher microhardness values than IPS e.max Press. Artificial saliva was associated with higher hardness values than the control condition, whereas citric acid was associated with lower values in both materials. The ethanol-exposed IPS e.max Press groups are therefore reported descriptively as non-measurable rather than included in inferential testing (Table 3; Figure 2b).

For surface roughness, two-way ANOVA showed significant main effects of ceramic material and FSL, together with a significant material × liquid interaction (material: *F*(1.40) = 756.86, *p* < 0.001; liquid: *F*(4.40) = 150.72, *p* < 0.001; interaction: *F*(4.40) = 63.98, *p* < 0.001). Overall, IPS e.max Press exhibited higher roughness values than CEREC Tessera, and roughness increased after acidic and alcoholic exposure. Descriptively, artificial saliva reduced roughness for IPS e.max Press relative to control, whereas citric acid and both ethanol concentrations produced marked roughness increases. In contrast, CEREC Tessera remained smoother across all conditions, although modest increases in roughness were observed after exposure to all FSLs relative to control (Table 3; Figure 2c). As shown in Table 4, the corresponding partial eta squared (ηp^2^) values indicated large material- and FSL-related effects for all three outcomes, with particularly strong effects observed for surface roughness and flexural strength.

## Discussion

This study compared pressed lithium disilicate (IPS e.max Press) and CAD/CAM lithium disilicate (CEREC Tessera) after exposure to FSLs. Factorial analysis showed significant effects of material and solution on flexural strength and surface roughness, with significant interaction effects, while microhardness analysis in the comparable shared conditions showed significant main effects of material and solution but no significant interaction. These findings reject the null hypothesis and indicate that fabrication route and material microstructure influence laboratory performance under chemical challenge.

### Flexural strength

CEREC Tessera demonstrated higher flexural strength than IPS e.max Press in the control condition and remained higher after artificial saliva and citric acid exposure (Table 4; Figure 2). These values are consistent with reports that CAD lithium disilicate may exhibit higher strength than pressed lithium disilicate, which is commonly attributed to differences in industrial processing control and the resulting flaw population [14]. This difference may be attributed to differences in fabrication route and flaw populations, as CAD/CAM blocks are produced under highly controlled industrial processing. In addition, Tessera has been described as an advanced lithium disilicate incorporating virgilite crystals within a zirconia-containing glass matrix, a formulation that may influence crack–flaw interactions and damage tolerance [26]. Post-processing also matters as manufacturer guidance recommends a glaze firing after milling to mitigate machining-related surface defects, which may contribute to improved surface integrity and strength retention. Variability among published findings (including studies reporting limited influence of food simulants on strength) likely reflects differences in exposure duration, cycling regimen, temperature, and the specific lithium silicate microstructures evaluated [27, 28].

### Microhardness

Microhardness was influenced by FSL exposure for both materials, with CEREC Tessera maintaining higher values than IPS e.max Press across comparable conditions. The reduction observed after citric acid exposure is consistent with the known susceptibility of glass-ceramic surfaces to acidic challenge, where hydrogen-ion activity may promote ion exchange, selective dissolution of the glassy matrix, and weakening of the superficial ceramic layer. As Vickers hardness reflects resistance to localized indentation, such surface-level changes can reduce the ability of the material to resist contact damage. The higher hardness values observed for CEREC Tessera may be related to differences in crystalline phase distribution, crystal size, and the zirconia-enriched glass matrix, although direct microstructural confirmation was beyond the scope of this study.

Notably, following ethanol exposure, the IPS e.max Press specimens exhibited a surface condition that prevented the formation of well-defined Vickers indentation margins under standardized criteria [24, 25]. This finding is best interpreted as an indication of altered surface integrity in the pressed specimens under the ethanol protocol, with the practical consequence that conventional Vickers microhardness could not be reported reliably for those groups. This observation should not be taken to imply that ethanol acts as a direct chemical etchant of lithium disilicate. Ethanol is generally considered less chemically aggressive to lithium disilicate glass ceramics than acidic media, while acids more directly affect the glassy matrix through ion exchange and selective dissolution processes [29]. Because indentation-based hardness is sensitive to surface flatness, surface roughness, glaze integrity, micro-chipping, and microcrack formation, complementary microscopic characterization would be needed to determine which of these mechanisms predominated in the ethanol-exposed pressed specimens.

### Surface roughness

Surface roughness increased after exposure to acidic and alcoholic media in both materials, while Tessera remained markedly smoother than IPS e.max Press across conditions (Table 4; Figure 2). Prior work similarly shows that acidic environments can increase roughness in glass ceramics by preferentially affecting the glassy phase and promoting surface irregularities [30–32]. Roughness is clinically relevant because it may facilitate plaque retention and staining and can act as a stress concentrator; therefore, the lower Ra values observed for Tessera under chemical challenge support careful finishing/polishing protocols and maintenance, particularly for patients with high erosive or dietary-acid exposure. At the same time, surface outcomes can be influenced by finishing steps (polishing versus glazing and the firing protocol), so roughness changes should be interpreted alongside the specific post-processing approach used.

### Mechanical context and implications

Citric acid challenge tends to be the most damaging because hydrogen-ion-driven ion exchange and selective dissolution of the glassy matrix can occur, and acid can diffuse through the glass network and along existing surface or bulk microcracks, accelerating crack propagation and lowering strength/reliability [29]. In contrast, ethanol (near-neutral pH) is typically less erosive for lithium disilicate glass ceramics and may have minimal impact on hardness compared with acidic solvents, supporting a more cautious interpretation than ‘ethanol etching’ of ceramics [1]. However, microscopy data in fully crystallized lithium aluminosilicates show ethanol-aged surfaces can present irregularities (scratches/craters), suggesting ethanol may accentuate surface defects or roughness rather than chemically etch the ceramic, which is relevant when explaining why indentation-based hardness readings can become unreliable when the surface is no longer flat and well-polished [13]. Recent evidence in advanced lithium disilicate systems also supports the role of thermal post-processing in surface flaw mitigation, as firing-related steps can improve strength and reliability by reducing machining-related defects [1], and refiring has been shown to close cracks more effectively in advanced lithium disilicate (Tessera) than in conventional lithium disilicate [12]. These observations provide direct microstructural support for the notion that post-milling thermal cycles, including glaze firing or refiring, can reduce the influence of machining-related flaws, although the magnitude of benefit is material-dependent and protocol-sensitive [13]. Finally, glazing can generate a homogeneous and glossy surface morphology, yet it may increase roughness depending on the firing/glazing approach, reinforcing the need to interpret roughness changes in light of both the chemical challenge and the finishing protocol [12, 33].

Clinically, these findings suggest that material selection and finishing protocol may be important when restorations are frequently exposed to acidic or alcohol-containing dietary challenges. The lower roughness and higher strength values observed for CEREC Tessera suggest favourable laboratory surface stability, although this should not be interpreted as direct evidence of superior clinical longevity. Intra-oral performance remains influenced by occlusal loading, fatigue, thermal cycling, saliva, biofilm, bonding, restoration geometry, and patient-specific habits.

### Limitations and future work

This *in vitro* study did not simulate intra-oral fatigue, dynamic pH fluctuations, or thermal cycling, and the chemical challenge was limited to selected media and a short conditioning period. The absence of SEM/AFM imaging restricts mechanistic interpretation of surface degradation and prevents direct correlation of roughness changes with specific microstructural features. Future work should incorporate thermomechanical cycling, longer and more diverse exposure regimens and microscopic characterization (SEM/AFM), and should directly compare clinically relevant finishing protocols (polishing, glazing, and refiring) to clarify how post-processing can mitigate chemically induced surface damage and improve mechanical stability.

## Conclusions

Within the limitations of this *in vitro* study, CEREC Tessera (CAD/CAM lithium disilicate) demonstrated higher flexural strength and microhardness and lower surface roughness than IPS e.max Press across the tested food-simulating conditions. Chemical challenge was associated with degradation in both materials, with the largest losses and post-exposure surface changes observed for IPS e.max Press, including ethanol-exposed groups where Vickers hardness could not be reliably measured. Although intra-oral conditions were not simulated, these findings suggest that fabrication route and resulting microstructure/surface integrity influence laboratory performance after chemical challenge; confirmation under thermomechanical cycling and microscopic surface characterization is needed before extrapolating to clinical longevity.

## Data Availability

The data that support the findings of this study are available from the corresponding author upon reasonable request.
